# A Web-Based Intervention to Address Risk Factors for Maternal Morbidity and Mortality (MAMA LOVE): Development and Evaluation Study

**DOI:** 10.2196/44615

**Published:** 2023-08-18

**Authors:** Alexis Dunn Amore, Abby Britt, Santiago J Arconada Alvarez, Morgan N Greenleaf

**Affiliations:** 1 Nell Hodgson Woodruff School of Nursing, Emory University, Atlanta, GA, United States; 2 Georgia Clinical and Translational Science Alliance, Emory University, Atlanta, GA, United States

**Keywords:** maternal mortality prevention, mobile technology, pregnancy, Black women, maternal, pregnant, mortality, web-based, utility, usability, mHealth, mobile health, algorithm, development, design, software, risk assessment, patient education

## Abstract

**Background:**

Maternal mortality in the United States is a public health crisis and national emergency. Missed or delayed recognition of preventable life-threatening symptoms and untimely treatment of preventable high-risk medical conditions have been cited as key contributors to the nation’s worsening mortality rates. Effective strategies are urgently needed to address this maternal health crisis, particularly for Black birthing populations. Morbidity and Mortality Assessment: Lifting Outcomes Via Education (MAMA LOVE) is a web-based platform that focuses on the identification of maternal morbidity and mortality risk factors.

**Objective:**

The purpose of this paper is to present the conceptualization, development, heuristics, and utility evaluation of the web-based maternal mortality risk assessment and educational tool MAMA LOVE.

**Methods:**

A user-centered design approach was used to gain feedback from clinical experts and potential end users to ensure that the tool would be effective among groups most at risk for maternal morbidity and mortality. A heuristic evaluation was conducted to evaluate usability and need within the current market. Algorithms describing key clinical, mental health, and social conditions were designed using digital canvas software (Miro) and incorporated into the final wireframes of the revised prototype. The completed version of MAMA LOVE was designed in Figma and built with the SurveyJS platform.

**Results:**

The creation of the MAMA LOVE tool followed three distinct phases: (1) the content development and creation of an initial prototype; (2) the feedback gathering and usability assessment of the prototype; and (3) the design, development, and testing of the final tool. The tool determines the corresponding course of action using the algorithm developed by the authors. A total of 38 issues were found in the heuristic evaluation of the web tool’s initial prototype.

**Conclusions:**

Maternal morbidity and mortality is a public health crisis needing immediate effective interventions. In the current market, there are few digital resources available that focus specifically on the identification of dangerous symptoms and risk factors. MAMA LOVE is a tool that can address that need by increasing knowledge and providing resources and information that can be shared with health care professionals.

## Introduction

Maternal mortality in the United States is a public health crisis and national emergency [1-3]. According to the Centers for Disease Control and Prevention (CDC), US maternal mortality rates (defined as the number of maternal deaths/100,000 live births) worsened from 2019 (20.1) to 2020 (23.8) [3]. Stark racial disparities persist in maternal mortality, with rates highest among Black women (55.3) as compared to White (19.1) and Hispanic (18.2) women. Missed or delayed recognition of preventable life-threatening symptoms and untimely treatment of preventable high-risk medical conditions have been cited as key contributors to the nation’s worsening mortality rates [3,4]. Effective strategies are needed to address this maternal health crisis, particularly for Black birthing populations [5].

The leading pregnancy-related causes of death among Black populations include cardiovascular conditions, hypertensive disorders, hemorrhage, and infection, conditions in which the timeliness of patient reporting and subsequent medical intervention are critical [3,4,6]. Additionally, mental and social conditions such as postpartum depression and psychosis as well as accidental death from violence, suicide, and opioid overdose increase the risk for self-harm; however, these factors have been largely overlooked in the evaluation of risk factors contributing to maternal death [6-11]. The risk for maternal mortality and poor health outcomes are also influenced by larger systemic, economic, and societal factors including lack of access to resources, racism, and bias [12-15].

Black women consistently report feeling ignored and unheard by providers when expressing concerns about health symptoms and the necessary resources to address them [16,17]. The development of interventions that not only improve the effectiveness of maternal mortality risk assessment but also provide ways to elevate the patient perspective and communication with providers may prove effective in combating the rising maternal morbidity and mortality rates. Technology-based health communications have proven to be effective tools to increase knowledge and improve communication activity among black populations engaged in mobile health (mHealth) research for people with other medical conditions [18,19]. Innovative strategies are needed that empower birthing people to overcome concerns of feeling unheard and disrespected in the birthing setting while ensuring that high-risk symptoms are addressed effectively.

There is limited research on the development, testing, and use of maternal mHealth apps that specifically address serious maternal morbidity and mortality risk for birthing people of color [20]. However, one study evaluated commercially available mHealth apps and the quality of the maternal health information content, app usability, and the inclusion of representation of people of color, who are those at the highest risk of serious maternal morbidity and mortality [20]. The authors screened over 300 mHealth apps, and only 25 met the inclusion criteria. The study found that most mHealth apps addressed peripartum behaviors but not health symptoms or risk factors, and most did not include adequate representation of birthing people of color [20]. Additionally, a meta-analysis of 15 randomized controlled trials implementing mobile technology to address perinatal mental and physical health showed moderate to large effect sizes in measured outcomes related to maternal physical and mental health and knowledge about pregnancy; however, there was no discussion on the impact on communities of color [20]. Other platforms focused on addressing maternal health issues in Black pregnant populations exist but are focused on topics related to weight management, gestational diabetes, and breastfeeding [21-24].

Taken together, these findings suggest that mHealth apps, when developed in collaboration with target demographic communities, appear to be an effective way to communicate information related to knowledge about maternal physical and mental health to birthing people. However, existing platforms do not currently address symptoms related to the high-risk conditions that contribute most to maternal morbidity and mortality, and those that do are not tailored toward communities of color, the population at greatest risk of experiencing adverse perinatal outcomes.

We conducted a market analysis in October 2021 looking at the availability and quality of evidence-based tools that provide customized symptom resources for Black female peripartum consumers. The market analysis was performed through a keyword search of the Apple App Store and Google Play Store, public websites, and PubMed. Our main findings included the Believe Her app (a CDC black mental health clinicians’ platform for peer-support and resource access) [25], the Irth app (crowdsourced reviews of health care providers from Black and Indigenous women of color) [26], and several web-based applications, such as Wolomi, Mahmee, and Mae (pregnancy and postpartum resources) [27-29]. These findings provide valuable insights into the market for evidence-based tools that cater to the specific needs of peripartum Black women consumers. While these platforms are important, it becomes apparent that there are a limited number of resources available that specifically address the needs of peripartum Black women consumers specifically related to risk factors for maternal morbidity and mortality [30]. Despite an increasing number of contributions to the field, there remains a critical gap in our understanding of how to best meet these unique health care needs of women of color during the peripartum period.

Morbidity and Mortality Assessment: Lifting Outcomes Via Education (MAMA LOVE) is a web-based platform that focuses on the identification of maternal morbidity and mortality risk factors, a feature not identified during our market search. The platform has been designed to address the leading medical conditions associated with maternal morbidity and mortality with an additional focus on mental health conditions and social determinants of health. Specifically, the platform provides the following: options for users to select symptoms associated with each category (physical, mental health, and social); feedback and recommendations on follow-up timelines for selected symptoms; tailored educational and resource links for selected symptoms; and a downloadable document of all physical, mental health, and social factors selected, which can be presented to health care providers as a communication tool. Tools that provide education for birthing people on perinatal urgent warning signs are needed to equip and empower them to seek care and communicate effectively with care providers. A key benefit provided by MAMA LOVE is the ability to tailor resources based on user-entered information, as evidenced by the market analysis. Given this capability, there may be regulatory implications in future versions of the tool. For example, Food and Drug Administration clearance would be required if the app were to make clinical recommendations based on the results of the survey.

The purpose of this paper is to present the conceptualization, development, heuristics, and utility evaluation of the web-based maternal mortality risk assessment and educational tool, MAMA LOVE. Specifically, in this paper, a detailed description of the content development process, feedback and end user recommendations, heuristic evaluation of the platform components, and final tool are presented along with a discussion about implications for future directions.

## Methods

**Figure 1.**
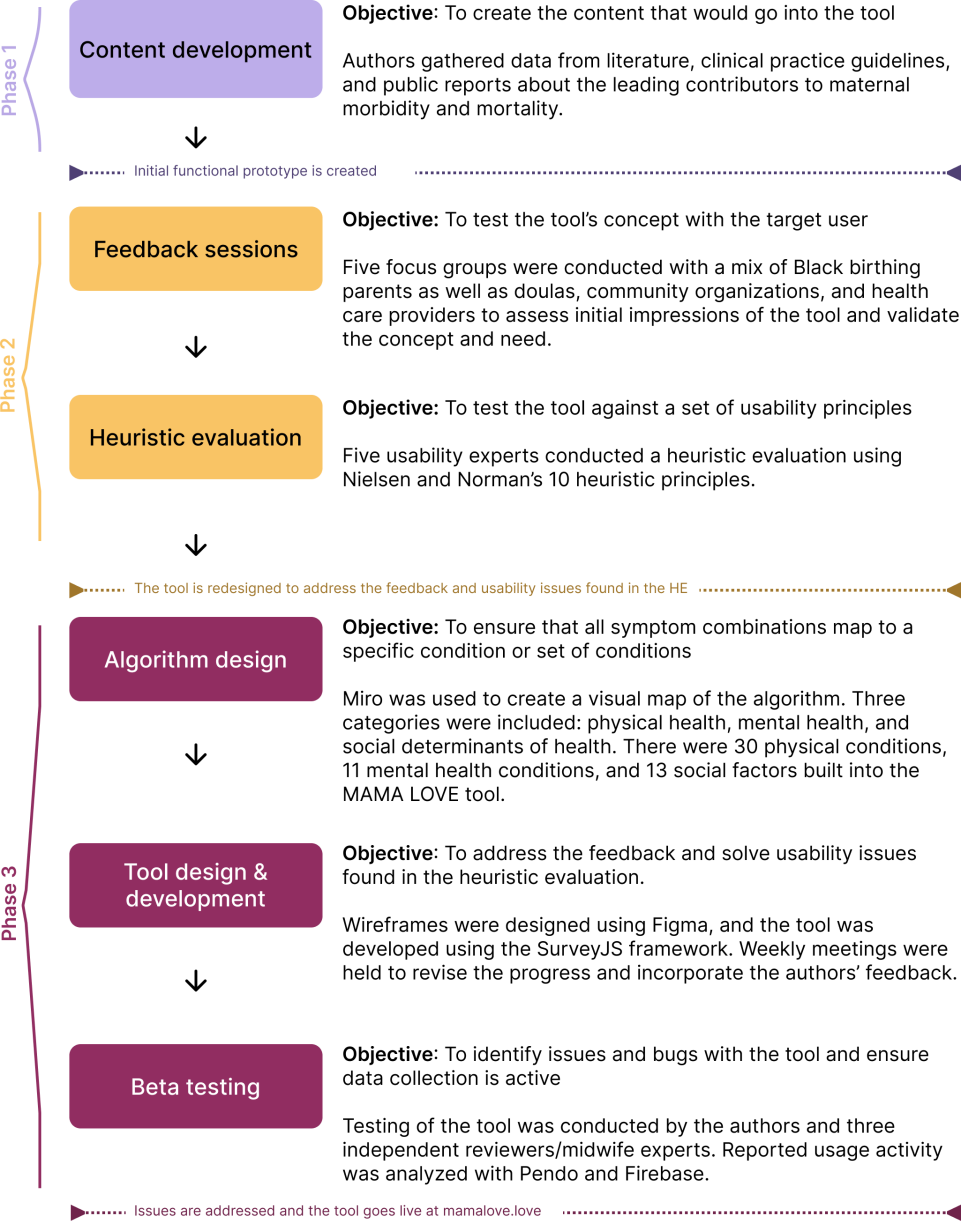
Flowchart of the study’s methodology. HE: heuristic evaluation; MAMA LOVE: Morbidity and Mortality Assessment: Lifting Outcomes Via Education.

The creation of the MAMA LOVE tool followed three distinct phases (Figure 1): (1) the content development and creation of an initial prototype; (2) the feedback gathering and usability assessment of the prototype; and (3) the design, development, and testing of the final tool. In this section, we further describe the methods for each of these phases.

### Phase 1: Content Development

#### Physical Indicators

The initial conceptualization of the tool was guided by information identified in clinical practice guidelines, publicly available data, and maternal mortality review reports related to the leading maternal health conditions contributing to morbidity and mortality, both in the United States and globally [7,8,31-33]. Epidemiologic-level data and factors identified in the literature were used to ensure that the most common high-risk physical conditions were included [3,7,9]. The CDC has leveraged administrative hospital discharge data and *International Statistical Classification of Diseases, Tenth Revision* (*ICD-10*) diagnosis and procedure codes to create a list of the 21 most common severe maternal morbidity conditions; these created the foundation for the symptom screening component of the app [33].

Additionally, the World Health Organization’s *The WHO Application of ICD-10 to Deaths During Pregnancy, Childbirth and the Puerperium: ICD-MM* report was consulted as a resource to ensure that the full range of most common causes of maternal death were incorporated into the physical screening portion of the tool [34].

#### Mental Health and Social Indicators

Health disparities are driven by multiple interrelated medical, social, and societal factors that promote susceptibility to disease [3,12-14]. Within the context of maternal mortality identification, medical conditions and their risks are well documented. However, less has been published on the relationship between maternal mental health conditions that increase the risk for self-harm and maternal mortality rates [35]. Additional content on maternal mental health conditions as well as social determinants were added given that the data on the leading causes of maternal death and severe illness include many of these factors as potential contributors [4,10-12,15]. The options available for selection by end users incorporate terminology that captures both the user experience as well as symptoms listed in clinical guidelines related to the conditions.

#### Resources

Educational resources were collected on each associated physical condition from reputable academic (ie, Mayo Clinic and University of California, San Francisco), governmental (ie, CDC), and nonprofit/community-based organizations (ie, Pre-Eclampsia Foundation, National Blood Clot Alliance, and Black Mamas Matter Alliance) to create customized educational reports based on the symptoms selected by the user.

#### Prototype

From the information identified in the literature, clinical practice guidelines, and public reports about the leading contributors to maternal morbidity and mortality, a prototype was built. This initial prototype of the tool (not shown) was developed via a collaboration between nurse midwifery researchers, community experts, and a software engineer at a private institution in Atlanta, Georgia. The initial prototype was developed with a third party with the guidance of the clinical authors over a period of 1 year. The format of the tool included an introduction to the MAMA LOVE platform; sociodemographic questions; symptom options for physical, mental health, and social factors; sample recommendations for a course of action options; and examples of resources that could be included. The goal of the prototype design was to validate whether the tool captured the most common symptoms and risk factors associated with high-risk perinatal conditions and to explore if the conditions and resources included in the tool were relevant to end users. Due to limitations in the software, the inability to make changes easily, and difficulty in addressing the heuristic evaluation results, a second tool was developed that allowed for more robust algorithm development and seamless transition between selected items, recommendations, and resources.

### Phase 2: Feedback and Usability Evaluation

#### Focus Group Sessions

Given the persistent racial and geographic disparities in maternal mortality risk, a robust approach inclusive of the community and social environment in which Black women reside may prove effective in ameliorating maternal mortality risk. As such, five focus group sessions (n=19) were conducted on the initial prototype with a mix of Black birthing parents (n=3) as well as doulas (n=6), community organization representatives (n=4), and health care providers (n=6) to assess initial impressions of the tool and to validate the concept. A rapid analysis of the focus group sessions was conducted to quickly identify key areas for feedback and revision of the tool [36]. The Emory University Institutional Review Board approved the study.

#### Heuristic Evaluation Methods

A heuristic evaluation is a qualitative assessment in which a usability expert measures a user interface against a set of principles to find usability issues. The heuristic evaluation was conducted on the initial prototype independently by five experts using Nielsen and Norman’s 10 Usability Heuristics, and the number of evaluators was determined using heuristic evaluation principles [37]. The experts responsible for conducting the heuristic evaluation consist of three graduates and two current students of the Masters in Human Computer Interaction program at the Georgia Institute of Technology, in collaboration with digital health developers at the AppHatchery at Emory University.

### Phase 3: Design, Development, and Testing

#### Algorithm Design

**Figure 2.**
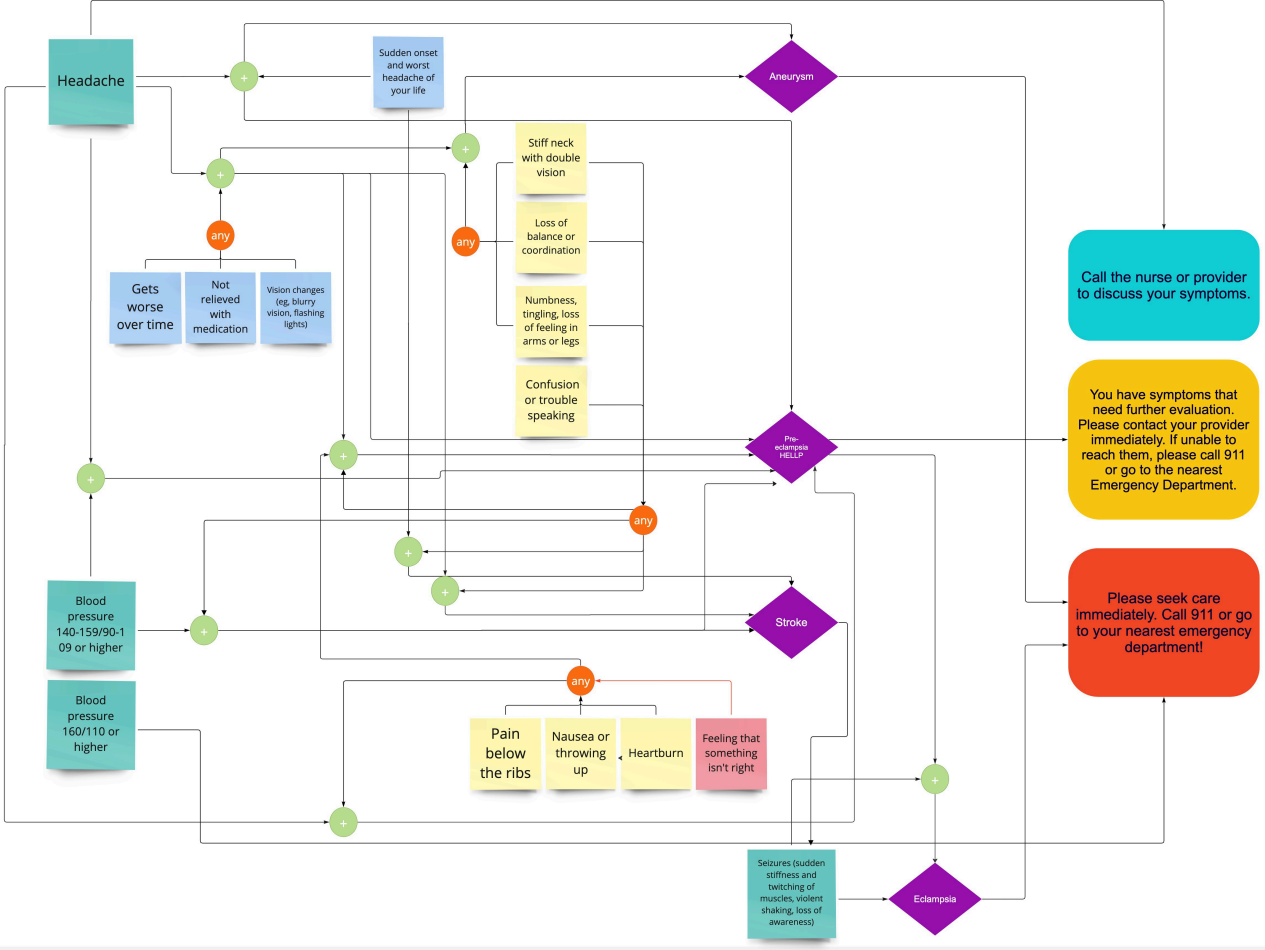
Visual representation of the algorithm for the head. Symptoms are represented in the turquoise, blue, and yellow sticky notes depending on the symptom category (main or additional). Conditions are in purple diamonds. The recommended course of action is outlined in the textboxes on the right-hand side. HELLP: hemolysis-elevated enzymes and low platelets.

The algorithm development followed an iterative approach in the translation from the clinicians to the developers. First, the clinician would list the symptoms related to a given condition and the suggested recommendation for the user. The developer partners would then review that representation and, with the clinicians, verify that the symptoms map properly to the condition. Common computer nomenclature was used to facilitate implementation and testing. Due to the algorithm’s complexity and many mappings between symptoms and conditions, a visual approach to organizing the information was used. Miro is a digital canvas tool that allows for real-time collaboration and facilitates mind mapping, which made it ideal for this use case. Symptoms in the algorithm were arranged head to toe and then mapped to select conditions (see Figure 2). Lastly, the language of the symptoms was carefully crafted to be understood by Black birthing parents, incorporating both the feedback from the focus groups as well as the authors’ experiences hearing patient questions in clinics.

#### Tool Design and Development

Limitations in the first prototype tool related to software, the inability to make changes easily, and difficulty in addressing the heuristic evaluation results prompted the development of a second and final tool that allowed for more robust algorithm development and seamless transition between selected items, recommendations, and resources.

The second tool was designed in Figma (Adobe), developed with the SurveyJS framework, and carried out by AppHatchery. AppHatchery is a National Institutes of Health–funded team part of the Georgia Clinical and Translation Science Alliance that creates patient-centered digital tools for health care through strategy, user research, design, software development, and clinical studies. The design and development of the tool took 6 months from August 2022 to January 2023, during which weekly meetings were held between the AppHatchery team and the clinical experts. Additionally, the collection of use metrics (Pendo.io) and survey responses (Firebase) was set up to validate the tool’s usability and intervention’s feasibility; this will be reported in a future paper.

#### Beta Testing

Once the tool was finalized and made available on MAMA LOVE, it was tested by the authors and 3 nurse midwifery experts from Emory University prior to public promotion of the platform. The aim was to find issues with the algorithm recommendations and to ensure the data collection was accurately capturing users’ behavior in the tool and their submitted responses. During this iterative testing process, inaccuracies were identified and corrected.

### Ethics Approval

Ethical considerations and safety were evaluated and approved via the Emory Institutional Review Board’s review process (IRB STUDY00002618).

## Results

### Phase 1: Content Development

#### Overview

The tool was primarily developed to screen for symptoms related to the most common conditions associated with severe maternal morbidity and mortality. From this list of conditions, clinician experts created symptom panels that map a user’s reported symptom experiences to a list of potential health conditions. Symptoms were arranged head to toe and then mapped to potential conditions that include the symptoms selected. Additionally, mental health conditions were included in the design prototype to further contextualize risk factors that may increase the risk for maternal morbidity or mortality. Recent findings indicate that mental and social conditions such as substance use disorder, suicide, and homicide are the leading contributors to serious maternal morbidity, particularly in the later postpartum period [11,35,38]. Overwhelmingly, mental health has been overlooked in the context of maternal mortality risk, but data suggest that more screening and attention to mental health are needed.

When ascertaining which social determinants of health to include in the tool, several modalities were leveraged. The first was expert clinician brainstorming sessions to identify the most common social determinants of health that have the potential to contribute to maternal morbidity and mortality. The search was then expanded to include, from an epidemiological perspective, the social determinants of health found to most commonly predict maternal morbidity and mortality at a large scale. The final conditions included in the tool were determined by the clinical authors in consultation with health care providers who participated in focus group sessions to assess the initial features of the tool. Additional feedback was gathered from other end users including community organizations, doulas, and Black women with experiences of high-risk morbidity conditions who participated in focus group sessions. The Area Deprivation Index was found to be a strong predictor of adverse perinatal outcomes [39-41]. Thus, the potentially modifiable components used to calculate this index were built into the tool, including housing, employment, and access to transportation. Finally, an extensive literature search was performed to further inform the selection of social determinants of health to ensure the most important factors were incorporated, including housing instability, intimate partner violence, rural location or transportation difficulties, and food insecurity [15,38,42-44].

#### Resources

Delayed reporting of concerning symptoms can result in ineffective and untimely management of potentially deadly conditions. The development of interventions that empower and educate pregnant populations to accurately recognize life-threatening symptoms, provide access to necessary resources, and promote effective communication with providers are needed to mitigate the maternal mortality risk among high-risk populations. It is essential that users are directed to reliable educational resources that consistently use the most up-to-date available evidence. Additionally, resources for mental health and other social determinants of health were included with a particular focus on including culturally focused content from groups like the Black Mamas Matter Alliance and Every Mother Counts organizations. Mental health resources were obtained primarily from Postpartum Support International (PSI), which is a leading nonprofit organization with a focus on perinatal mental health conditions [45]. In addition to educational information on perinatal mood disorders, users can access a PSI-sponsored 24-hour emergency crisis hotline for immediate support and a directory with mental health providers in their area. State- and national-level resources to address food insecurity, housing instability, insurance enrollment information, and transportation resources are also included.

### Phase 2: Feedback and Usability Evaluation

#### Focus Group Sessions

To ensure that MAMA LOVE would be effective, feedback on the first prototype was sought from Black birthing people and community stakeholder experts (comprised of doulas, community organizers, and health care providers). Key aspects identified in the rapid analysis of the focus group sessions included simplification of the terminology used across the platform, incorporation of examples to describe conditions, features to show the end user how far along they are in the platform, grouping of demographics questions, use of more images and fewer words, and expansion of resources lists to include more content from diverse groups that serve Black pregnant populations. A more robust thematic analysis of the focus group sessions is reported elsewhere (manuscript in submission). These preliminary findings were used to inform the development of the second tool.

#### Heuristic Evaluation Results

**Figure 3.**
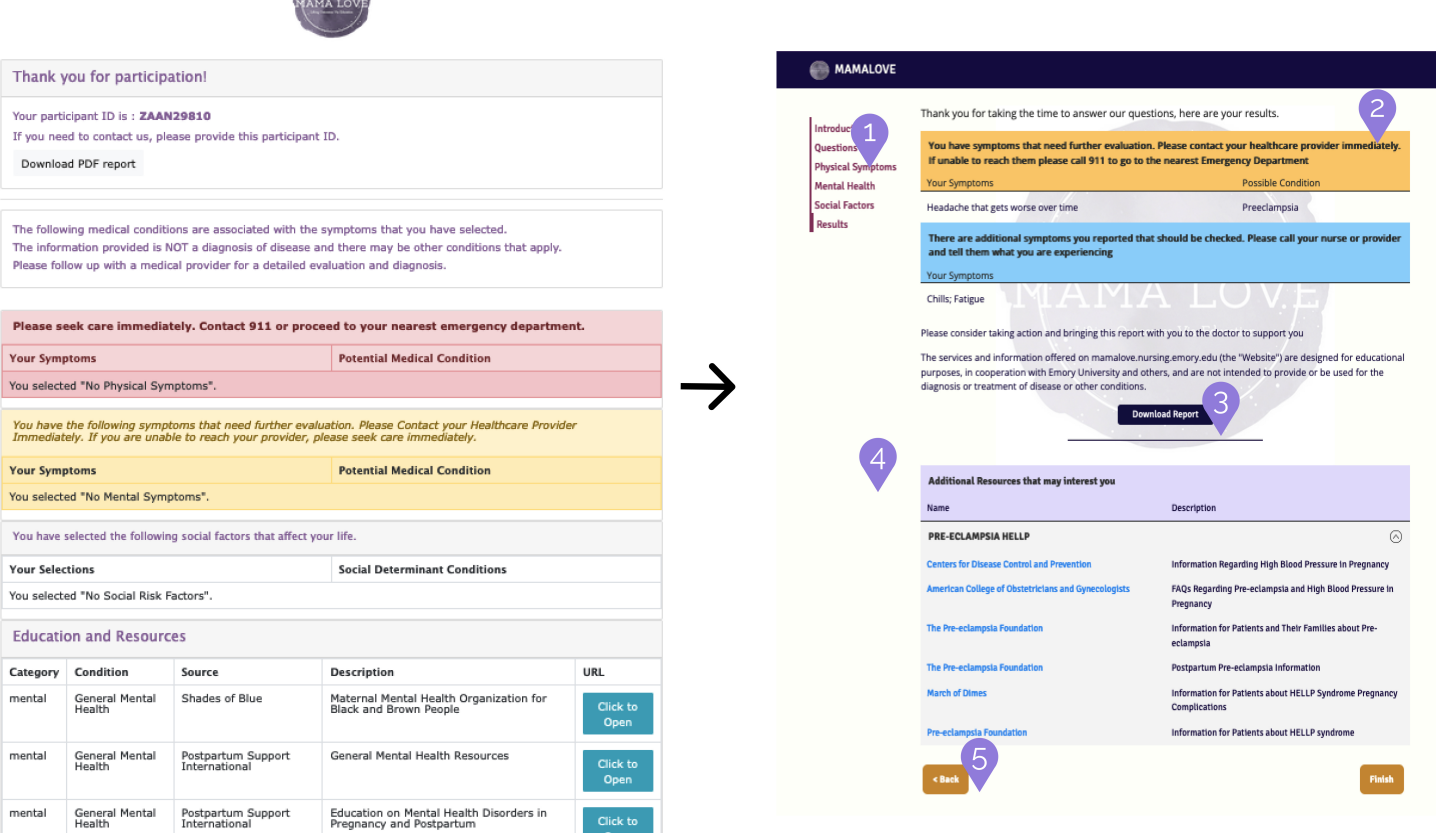
Comparison of the results page after addressing issues described in the heuristic evaluation. Left: old; right: new.

Following a heuristic evaluation with 5 usability experts, a total of 38 issues were found in the initial prototype of the web tool. Sample comments for each usability factor related to the website are reported in Table 1. The newer version of the tool implemented changes to tackle the major usability issues such as navigation (allowing the user to move freely back and forth through the survey), mobile responsiveness (text and content should scale accordingly to the device type being used), and the consistent use of standard button and interaction paradigms throughout the tool. For the purposes of providing context, a visual example of the changes implemented between the prototype and the final tool is shown in Figure 3.

**Table 1.** Heuristic evaluation results and changes.

Heuristic principle	Sample quote	Design alterations
Visibility of system status	“After selecting symptoms on the cards themselves, the user doesn’t see what those selections were.”	Text with a time estimate for completion of the survey was included. The section the user is on was highlighted on a navigation menu.
Match between system and the real world	“It might be better to put some illustration of the body parts.”	Images were not included as part of the new design due to time constraints on the construction of the second version of the tool.
User control and freedom	“Users are not allowed to go back to change their answer.”	A back button on all pages and a navigation bar were incorporated to allow user mobility through the different sections of the tool.
Consistency and standards	“After selecting a symptom type, the questionnaire changes style into a popup window, and each category (which was not clear they were categories vs. answers) is scrollable into expanded choices- different from the first half.”	The format of web elements such as hyperlinks, buttons, and banners followed standards. A visual style was applied to the tool to improve consistency from page to page.
Help users recognize, diagnose, and recover from errors	“Clicking save & continue button without clicking the checkbox doesn’t prompt the user with any messages nor visual feedback of what is missing.”	An error message pops up indicating to the user that they must select an answer to proceed.
Error prevention	“On mobile, the selection boxes are really close together.”	Larger checkboxes or tappable targets were used. Allowing the user to go back to verify what symptoms they have selected can prevent a wrong assessment.
Recognition rather than recall	“Unclear that if you are answering age, if it’s your own or the person you are filling this out for. The language for the questions doesn’t remind the user to answer for another person if this was their choice.”	User selections are saved from page to page so that going back does not require them to remember what they selected.
Flexibility and efficiency of use	“It asks what type of delivery the user had, although I selected ‘No’ in the question about giving birth in the last year.” “Symptoms related to a cesarean infection showed up although I did not select cesarean birth.”	Conditional logic was applied so that questions or symptom selections that are irrelevant for the user do not appear. A tappable area was added to the selection text in addition to the checkbox or radio button to improve speed of use.
Aesthetic and minimalist design	“Pages are not responsive for mobile, text doesn’t scale, and information is very hard to read.” “All questions have the label Question.”	Mobile responsiveness was incorporated to allow text and content to scale properly. Redundant text and information was removed to reduce visual cognitive load.
Help and documentation	“It is unclear what the user should do on the category selection page. The ‘Save & Continue’ button appears only when you select items in each category. This is not obvious to the user.”	Instructions were included throughout the tool to support the users in areas where confusion may arise, such as symptom selection.

The annotations in Figure 3 indicate the changes that were made to the updated version of the tool. These changes aimed to improve the user experience and make the tool more user-friendly:

1. The addition of a navigation menu allowed users to easily navigate to any section of the tool they want to revisit. This provides users with more freedom and improves the visibility of system status by highlighting the section they are currently on.
2. Removal of redundant elements, such as empty content, and the reorganization of the information architecture helps visually prioritize urgency. This means that potential conditions are listed first, so users can immediately see what is important without having to scroll.
3. Changing the format of the download button and presenting it by itself can help users locate the button and avoid confusion over its purpose.
4. The resource section only includes necessary information, and URL links are formatted following web standards to improve user understanding and expectations of their behavior.
5. The addition of a back button allows users to go back and change their choices if they wish, while the finish button ensures that the user knows there is no more content to interact with.

Overall, the changes made to the tool were designed to make it more user-friendly and improve the user experience. The annotations on the figure provide a clear visual representation of the changes that were made, making it easy for readers to understand how the updated version of the tool differs from the original version. However, there were more changes that were implemented to improve usability, such as saving user selections on previous pages, using consistent colors and standards, increasing the tapping targets so clicking is easier, reducing the amount of text on the screen to the strictly necessary, and making the website mobile responsive.

### Phase 3: Design, Development, and Testing

#### Algorithm Design

**Figure 4.**
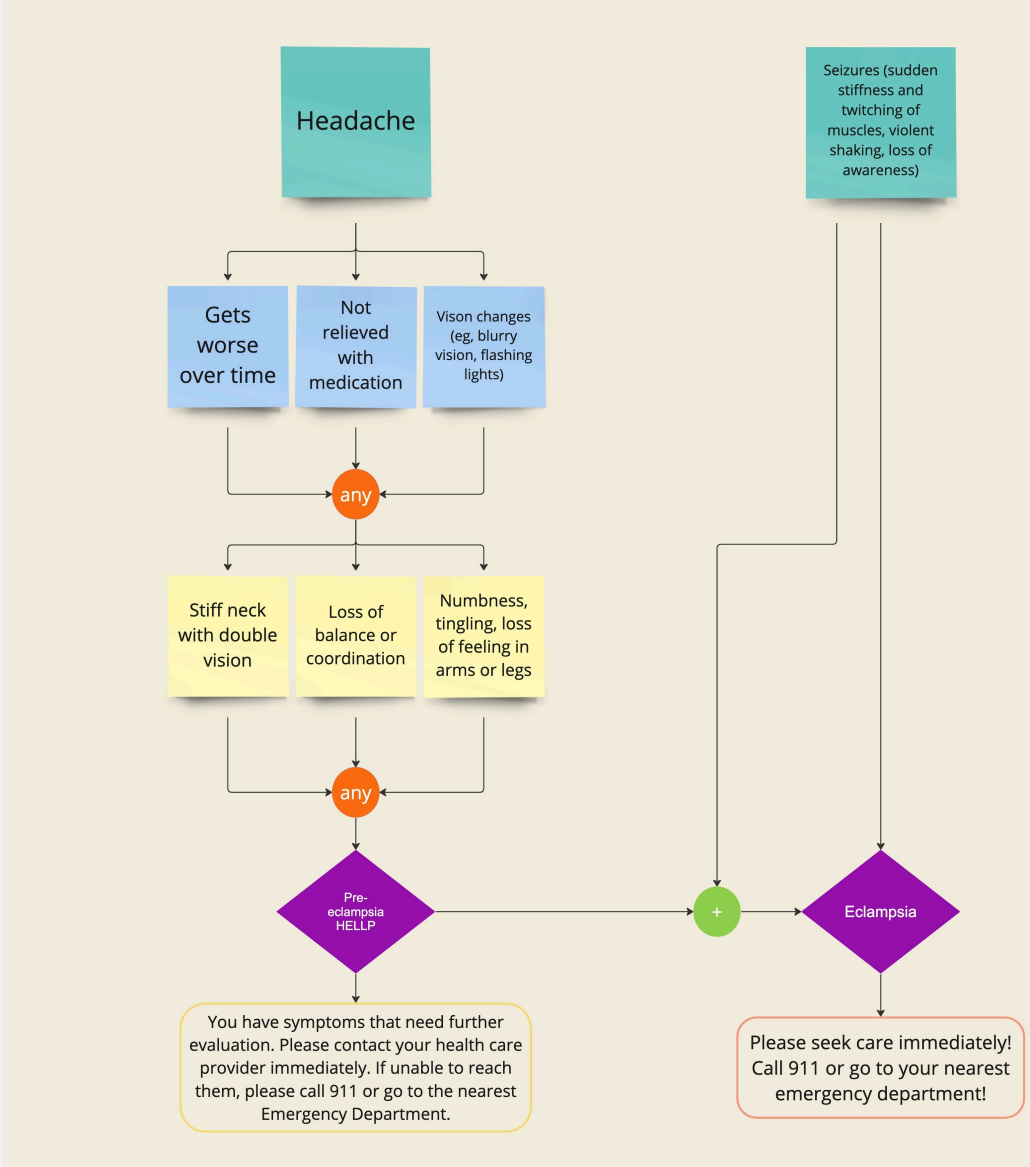
An example of a user with symptoms associated with pre-eclampsia (not able to measure blood pressure). If a user indicated they have a headache that gets worse over time and they are having confusion or trouble speaking, the tool would predict they have pre-eclampsia. Additionally, if the user is having seizures, the tool will predict eclampsia. It is worth noting that if the user is having seizures, the tool will predict eclampsia immediately without needing the other symptoms. Depending on the predicted condition, the tool would provide a recommended course of action tailored to the severity of the condition (ie, pre-eclampsia: contact your health care provider; eclampsia: go to the hospital). However, a user could arrive at these conditions through different symptom paths (refer to other paths in the diagram and to Figure 1 for an expanded view of more symptoms). HELLP: hemolysis-elevated enzymes and low platelets.

**Figure 5.**
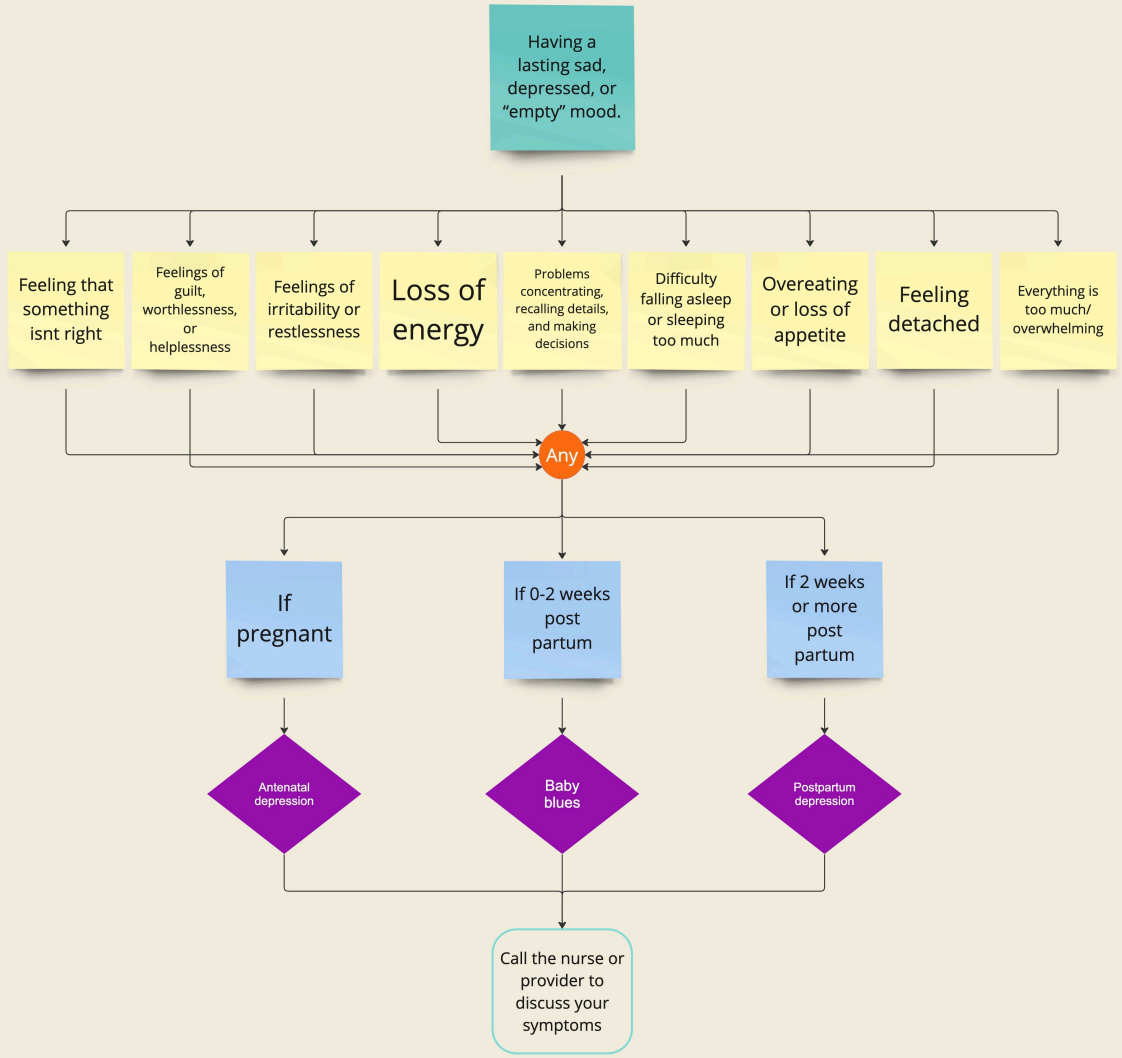
An example implementation of the algorithm for mental symptoms. If a user indicated they have a lasting sad, depressed, or “empty” mood; feelings of irritability; or restlessness while they are pregnant, the tool would predict they have antenatal depression. Alternatively, if the user is 0-2 weeks post partum, the tool will predict baby blues, and if greater than 2 weeks post partum, it will predict potential postpartum depression. Depending on the predicted condition, the tool would provide a recommended course of action tailored to the severity of the condition; in this case, all three possible conditions suggest calling the nurse or provider to discuss the symptoms.

**Figure 6.**
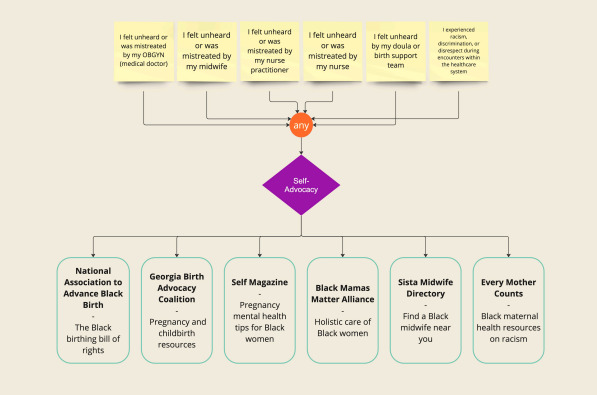
An example implementation of the algorithm for social risk factors. If a user indicated they felt unheard or mistreated by their OBGYN (medical doctor) the tool would recommend them to explore a list of self-advocacy resources including but not limited to the Black Mamas Matter Alliance, which is a holistic care for Black women resource. Depending on the predicted need (self-advocacy, prenatal resources, etc) the tool would provide a list of recommended resources. In this case, all social risk factors displayed map to a recommended need for self-advocacy. OBGYN: obstetrics and gynecology.

The tool determines the corresponding course of action using the algorithm developed by the authors. Following the decision to create a second tool, the algorithm to determine the corresponding course of action was developed between the clinician and developer partner. Examples of algorithms from each of the three categories included in MAMA LOVE are outlined in Figures 4, 5, and 6. Figure 4 outlines the pathway that could lead to pre-eclampsia or eclampsia in a setting where a client would not have access to measure their blood pressure. Figure 5 outlines the pathway related to antenatal or postpartum depression. Figure 6 outlines experiences of racism and mistreatment in the perinatal setting and the pathway to self-advocacy resources.

#### Final MAMA LOVE Design and Development

**Figure 7.**
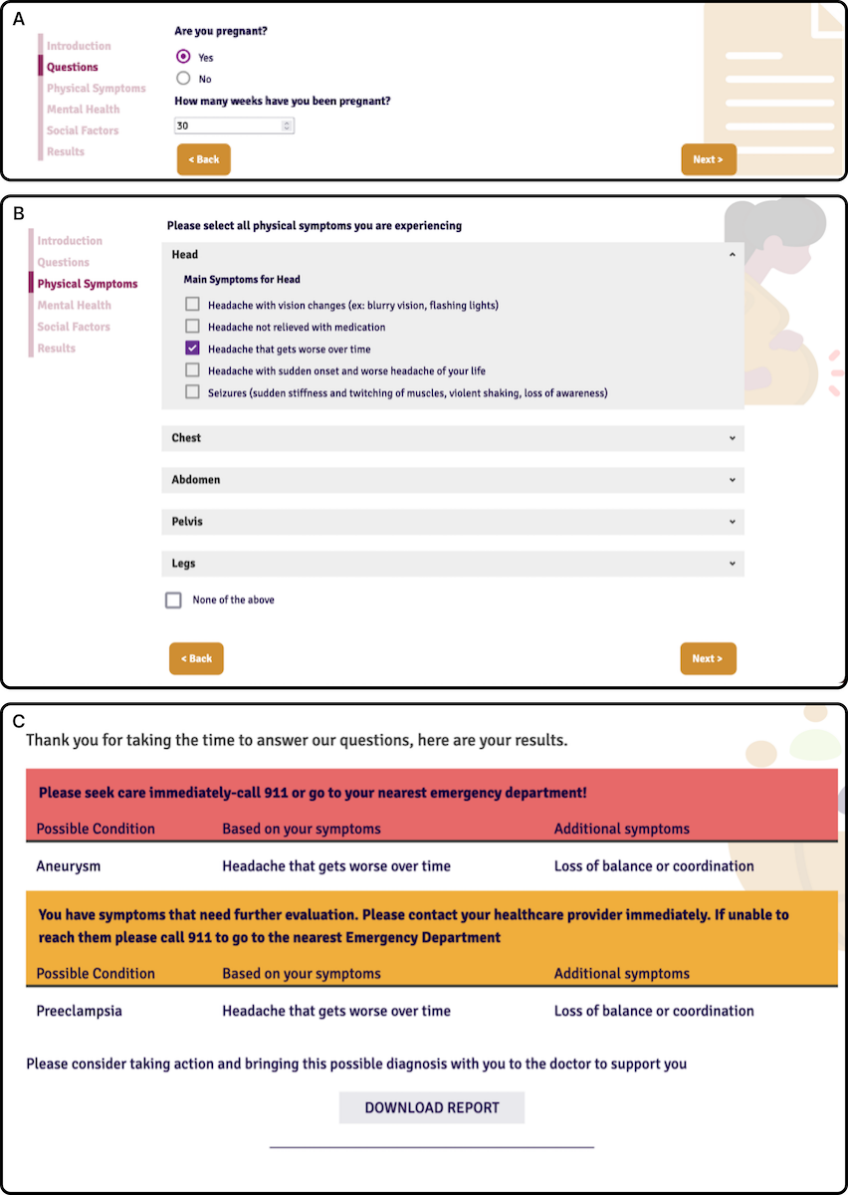
Screenshots of the final version of Morbidity and Mortality Assessment: Lifting Outcomes Via Education (MAMA LOVE). (A) Stage of pregnancy. (B) Physical symptoms selection page. (C) Results page.

MAMA LOVE is a web-based tool designed to support Black birthing populations by providing guidance and resources on the leading high-risk conditions. The tool follows a survey structure (Figure 7). It begins with a brief introduction explaining the purpose of the tool, questions, and information being collected. It then moves on to a series of simple questions to collect demographic information and the stage of pregnancy or postpartum course. The user can then select whether they have any specific physical symptoms, mental health symptoms, or social risk factors. The final page includes a recommended course of action based on the user’s selected symptoms as well as a list of recommended resources based on reported risk factors.

#### Beta Testing

Prior to public release, the research team of midwives and AppHatchery experts encountered several issues related to the mapping of symptoms to conditions. For example, the algorithm for symptoms that link to pre-eclampsia also includes those that overlap with the stroke algorithm. During the evaluation, it was noted that the end output was only identifying stroke as a potential condition. The team identified that the language used by the lead midwife researcher on the visual algorithm platform was being read differently by the design team. Once this difference was identified, the issue was corrected, thus resulting in the desired end conditions appearing in the output. Each section of the tool was carefully evaluated and tested in this manner.

## Discussion

### Overview

There is limited research on the development, testing, and use of maternal mHealth apps that specifically address serious maternal morbidity and mortality for birthing people of color. Of those evaluated in a recent meta-analysis of 15 randomized controlled trials of mobile technology addressing peripartum physical and mental health behaviors, none specifically focused on the identification of symptoms and risk factors for morbidity and mortality in the general population nor among populations of color [30].

MAMA LOVE is designed to address the leading physical, mental health, and social risk factors that increase the risk for maternal morbidity and mortality. The leading pregnancy-related causes of death among Black women include cardiovascular conditions, hypertensive disorders, hemorrhage, and infection, conditions in which the timeliness of patient reporting and subsequent medical intervention are critical, as many of these deaths are deemed preventable [7]. The additional focus on mental health and social determinants of health adds a layer of screening that contextualizes risk and highlights the importance of evaluating the individual lived experience within the context of “risk.” The final algorithms used in the creation of the MAMA LOVE platform incorporate these concepts in a fashion that is designed to be usable and effective for the end user. As such, this tool would be generalizable and useful, particularly among at-risk populations.

Resources are included in the tool that direct users to well-known, community-led, and state- and national-level academic, governmental, and nonprofit resources. However, there is a need for more robust culturally tailored educational and social support resources. There was an effort to include resources from community groups caring for people of color; however, those resources were not plentiful. The need for culturally designed resources is particularly important for those most at risk for severe maternal morbidity and mortality. Using this cultural perspective, we worked with the development group during the design process to ensure that the imagery, language, and functionalities of the tool were designed with the end user in mind.

### Comparison With Prior Work

There is limited research available regarding the use of mHealth apps and their effectiveness in mitigating maternal mortality risk. As previously discussed, there are apps available that provide quality content around health behaviors in pregnancy. However, they do not adequately represent people of color. It has been shown that Black women are willing to use their smartphones to access health promotion content and to participate in mHealth research [18]. Although more than 300 mHealth apps are available, most offerings do not provide adequate evidence-based maternal health information and are not reflective of women of color [20]. We expect that a platform providing immediately accessible, accurate, timely, and culturally appropriate information will be uniquely advantageous when used for the preventive care of Black women [5], as has been shown in Black women experiencing other medical conditions [19,46,47]. As such, the addition of MAMA LOVE fills a unique gap in that it is a community-designed platform specifically focused on the mitigation of maternal mortality risk.

While other studies detailed the development and testing of mHealth apps for pregnant people of color, they focused on topics other than serious maternal morbidity and mortality, such as postpartum fitness and weight loss (BeFAB) [24], physical activity and weight control during pregnancy [23], gestational diabetes (SweetMama) [21], and breastfeeding (KULEA-NET) [22]. The developers of these mHealth apps used a similar approach for the development of their app by reviewing current mHealth app technology and evidence-based best practices for their topic areas to create an initial prototype, conducting formative research with women in the potential user demographic or health care providers, and incorporating their feedback into the final tool. These studies provide support for the mHealth app development pathway used by MAMA LOVE, with an initial design based on current evidence and subsequent iterative feedback sessions with pregnant people of color and health care providers as an effective way to tailor an mHealth app for communities of color.

### Limitations

The tool is limited in its scope as it identifies symptoms or social conditions after they have already occurred. In the quest to prevent maternal morbidity and mortality, this may be too late. There is a need for a tool or resource that captures data and information in real time so that interventions can be instituted earlier in the pregnancy or postpartum period. Additionally, the resources that are included in the tool will have to be continually revised to ensure accuracy and relevance to the population. Additionally, the tool has been developed by researchers and app developers in the southeastern United States; as such, the types of resources included were naturally influenced by this geographic lens.

The development group responsible for building the tool consisted of individuals with diverse backgrounds, including in product management, design, and development. The team had prior experience working on other technology projects aimed at African American minorities, which provided a strong foundation for their work on this tool. The group was composed of individuals from African Black, Hispanic, and White backgrounds, which helped ensure a broad perspective and inclusivity during the design and development process.

In addition, the group leveraged specific design methodologies to navigate cultural values and paternalism during the development of the tool. These methodologies included incorporating common visual styles and themes from other tools aimed at Black birthing parents, such as using visuals of birthing parents throughout the tool. The team also sought to avoid paternalistic and critical language in the tool, which could be perceived as condescending or dismissive. By taking these measures, the development group aimed to create a tool that was culturally sensitive and inclusive while also providing accurate and useful information to users.

Despite the outcomes of the heuristic evaluation and design changes implemented, it is important to acknowledge the limitations. Although the conceptualization of the project and creative design of the tool was led by a Black researcher, the evaluation of the tool was conducted by a team of designers who were not specialized in website design for Black birthing parents. While the team had experience in evaluating and designing digital tools, they may not have been fully attuned to the specific cultural and experiential needs of this population. As such, it is possible that certain design issues or cultural considerations were not fully captured by the evaluation, and additional research by specialized user experience–specific experts may be warranted to further refine the tool.

### Conclusions

Maternal morbidity and mortality is a public health crisis needing immediate effective interventions. Comprehensive models and strategies that improve the ability of at-risk groups to better identify their risk are needed. MAMA LOVE seeks to empower pregnant populations with knowledge and resources to encourage self-advocacy, particularly among Black populations who have the greatest risk of dying during the perinatal period. Given that many pregnant populations have identified that they often do not feel heard or are not taken seriously when presenting with concerning symptoms, MAMA LOVE is a tool that has the potential to address that need by increasing knowledge and providing information that can be shared with health care professionals.

## Abbreviations

CDC: Centers for Disease Control and Prevention
*ICD-10*: *International Statistical Classification of Diseases, Tenth Revision*
MAMA LOVE: Morbidity and Mortality Assessment: Lifting Outcomes Via Education
mHealth: mobile health
